# Dentistry in China

**Published:** 1894-01

**Authors:** J. G. Kerr

**Affiliations:** Canton, China


### Dentistry in China.

BY J. G. KERR, M.D., CANTON, CHINA.

Abstract of a paper read before the Ohio State Medical Society, June, 1893.

In the department of dentistry the Chinese have, strange to relate, anticipated by centuries the profession in Europe and America in the insertion of artificial teeth. Utilizing the femur of an ox, and sawing a circle of half or three-quarters of an inch from the shaft, a section of this circle is used sufficient to fill the vacant space in the mouth. The section of bone is then dressed with a file, so as to imitate the teeth to be replaced, and through holes drilled in each end, copper wires are passed to fasten it to the adjoining teeth. These artificial teeth are designed more for good looks than for purposes of mastication, and since the cost of inserting three or four teeth amounts to about twenty-five or thirty cents, this means of remedying uncomely defects is within the reach of all.

American and English dentists of high standing have practiced their profession in Hong Kong, Shanghai, and other cities open to foreign commerce, and have employed Chinese young men to assist in the mechanical part of the work. With the talent for imitation for which the race is noted, these young men have not been slow to avail themselves of the opportunity of learning the more delicate parts of the work performed by the dentist himself. A number of these young men have become successful practitioners among their own countrymen, and with foreign instruments and material, are superseding the crude and unsatisfactory work of the native artists. They have not yet attained to the skill in the treatment of diseases of the mouth which requires scientific knowledge, but that will come in time.

The theory that toothache depends on the presence of worms in decayed teeth is universally believed, and is demonstrated by a process peculiarly Chinese, and which was investigated some years ago by Dr. Rogers, a dentist of Hong Kong, and myself. The native operator holds back the lips with a wooden spatula while he works around the offending tooth with a pointed instrument until there is a flow of saliva and blood; adroitly turning the spatula and placing the other end in the mouth, a piece of delicate paper attached to one side is moistened by saliva and the worms, confined under it, are liberated, and become mixed up in the bloody saliva. With a pair of forceps the operator picks them out and satisfies the patient.

